# Amplify-and-Forward Cooperative Diversity for Green UWB-Based WBSNs

**DOI:** 10.1155/2013/785840

**Published:** 2013-11-06

**Authors:** Heba Shaban, Mohamad Abou El-Nasr

**Affiliations:** ^1^Electronics and Communications Engineering, Arab Academy for Science, Technology & Maritime Transport (AASTMT), Alexandria 1029, Egypt; ^2^Computer Engineering, Arab Academy for Science, Technology & Maritime Transport (AASTMT), Alexandria 1029, Egypt

## Abstract

This paper proposes a novel green cooperative diversity technique based on suboptimal template-based ultra-wideband (UWB) wireless body sensor networks (WBSNs) using amplify-and-forward (AF) relays. In addition, it analyzes the bit-error-rate (BER) performance of the proposed nodes. The analysis is based on the moment-generating function (MGF) of the total signal-to-noise ratio (SNR) at the destination. It also provides an approximate value for the total SNR. The analysis studies the performance of equally correlated binary pulse position modulation (EC-BPPM) assuming the sinusoidal and square suboptimal template pulses. Numerical results are provided for the performance evaluation of optimal and suboptimal template-based nodes with and without relay cooperation. Results show that one relay node provides *~*23 dB performance enhancement at 1*e* − 3 BER, which mitigates the effect of the nondesirable non-line-of-sight (NLOS) links in WBSNs.

## 1. Introduction

Diversity is one of the prominent techniques for fading mitigation. One of the most effective diversity techniques is space diversity, where multiple antennas are adopted at the transmitter, receiver, or both ends. Recently, cooperative diversity has been proposed as a promising candidate for wireless communication systems. It achieves the performance of multiple-input-multiple-output (MIMO) systems through the use of neighboring relays for relaying the signal between the source and destination nodes. Accordingly, the destination node receives multiple independent signals and achieves diversity without using multiple antennas [1–3]. 

On the other hand, ultra wideband (UWB) has received increased attention from researchers as well as industry as a promising technology for short-range communications. In particular, UWB is characterized by its ultra wide bandwidth, −10 dB bandwidth greater than 500 MHz, as well as robust performance in dense multipath environments. Nakagami-*m* fading demonstrates many types of fading channels and was proposed by the IEEE 802.15.4a model for ray-arriving clusters [4–7]. In [8], based on actual on-body measurements, Nakagami-*m* channel was used to model actual on-body UWB channel and the average *m* parameter from different scenarios was found to be 1.5. 

One of the challenges associated with UWB systems, especially UWB-based wireless body sensor networks (WBSNs), is the design of an efficient receiver. Particularly, one of the main requirements of WBSNs is low-power consumption. WBSNs include medical and nonmedical applications, such as health monitoring, biomedical applications, gaming, and computer animations [9, 10]. However, in the medical applications, data is highly sensitive to errors. Thus, it is desirable to use optimum coherent receivers, which requires the template pulse be matched to the incoming pulse which is in turn hard to implement and power consuming. On the other hand, suboptimal noncoherent detectors are available, but their performance is highly suboptimal.

Another challenge associated with the design of efficient WBSNs is the highly degraded performance of non-line-of-sight (NLOS) links. Recent studies showed that for NLOS on-body communications, up to 20 dB attenuation occurs due to the presence of a body limb that blocks the line-of-sight (LOS) path between transmit and receive nodes [10, 11]. In order to guarantee freedom of movement in on-body nodes of wearable BSNs, the employment of diversity techniques would not be the ultimate solution. Thus, cooperative communication is a promising candidate for BSNs to mitigate the attenuation caused by body limb obstruction in NLOS links. However, the trade-off between power consumption and BER performance remains as an important issue.

Greening of networking refers to reducing unnecessary energy expenditure in the network. A real challenge in green networking is to save energy consumption (or equivalently power consumption) while maintaining high network performance [12, 13]. Consequently, green communications require efficient alternatives that save energy/power with a minimal performance loss. Thus, low-power alternatives that save energy/power traded for performance degradation, such as noncoherent detectors, are not considered appropriate candidates for green networking. 

Recently, low-power suboptimal template-based coherent detectors have been proposed in the literature as a low-power alternative for optimal coherent detectors that provide an approaching performance to the corresponding optimal template-based coherent detectors [10, 14–16]. These templates include windowed sinusoidal and square pulses [12, 17]. The power consumption of an impulse radio UWB (IR-UWB) analog correlation receiver that uses a real suboptimal sinusoidal template for a signal bandwidth of 500 MHz and 2 Mbps bit-rate is *≈*16.8% of the power consumed by the corresponding digital receiver structure for the same design parameters. This value is also smaller than the power consumed by the corresponding noncoherent transmitted-reference (TR) receiver, also with the same design parameters [15, 18].

In this paper, we study the performance of amplify-and-forward (AF) cooperative diversity with multiple relays (*L*) that employ suboptimal sinusoidal and square pulse templates in independent nonidentical Nakagami-*m* channels for green WBSNs. In our analysis, we use the moment-generating function (MGF) of the signal-to-noise-ratio (SNR) and provide approximation of the performance based on the analysis introduced in [19]. In our analysis, we consider equally correlated BPPM (EC-BPPM). 

## 2. System Model and Design of Template Pulses

**Figure 1:**
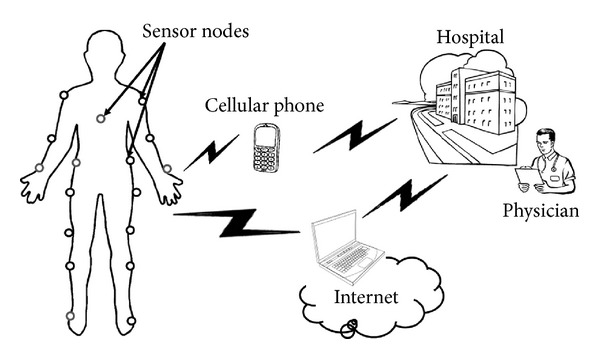
Schematic diagram of wireless body sensor networks (WBSNs).

In this section, we present the optimal and suboptimal pulses and their parameters that will be used at the receive nodes of WBSNs.  Figure 1 shows a schematic diagram of WBSNs. The studied suboptimal pulses include sinusoidal and square pulses. In the system model, there are a source node (*S*) and destination node (*D*) that communicate over a channel that exhibits flat Nakagami-*m* fading. Between the source and destination nodes, there are a number of cooperative nodes that relay the signal to the destination node. We assume using maximal ratio combiner (MRC) at the destination node to combine the different incoming replicas of the signal. At the destination node, the receiver fingers are based on coherent detection, where a template pulse is correlated with the incoming signal. The most commonly used pulses for impulse-radio UWB (IR-UWB) transmission are the derivatives of the Gaussian pulse, also referred to as Gaussian monocycles. For optimal detection, the template pulse should perfectly be matched to the received pulse.

The *n*th-order Gaussian pulse *ω*_*n*_(*t*) in terms of *σ* ^2^ = *T*_*p*_/2*π* and the pulse duration *T*_*p*_, have the following form [14]:

(1) $$\begin{array}{c} \omega_{n}\left(t\right)=\frac{d^{\left(n\right)}}{dt^{n}}\left(\frac{1}{\sqrt{2\pi \sigma^{2}}}e^{-t^{2}/2\sigma^{2}}\right). \end{array}$$

Assuming a correlation receiver, the optimal template *v*(*t*) should be matched to the received pulse. Typically, the pulse order should be chosen to meet a specified Federal Communication Commission (FCC) system's allowable emission limits. Typically, for correlator receivers, the design parameters of optimal and suboptimal templates should be chosen to maximize the output SNR [14] as follows:

(2) $$\begin{array}{c} \text{SNR}=\frac{E_{s}}{N_{0}}\frac{R_{pv}^{2}\left(\tau_{e}\right)}{R_{vv}\left(0\right)}, \end{array}$$

where *E*_*s*_ is the bit energy, *N*_0_ is the noise PSD, *R*_*pv*_(·) is the normalized cross correlation of the received pulse and the template waveform, *τ*_*e*_ is the timing error, and *R*_*vv*_(·) is the normalized autocorrelation of the template pulse. In case of optimal pulse, *R*_*pv*_(·) is equal to the pulse autocorrelation *R*_*pp*_(·). The autocorrelation of the optimal pulse is given by

(3) $$\begin{array}{c} R_{pp}\left(\tau \right)=\frac{1}{E_{p}}\int_{-\infty }^{\infty }p\left(t\right)p\left(t-\tau \right)dt. \end{array}$$

### 2.1. Suboptimal Sinusoidal Templates

Suboptimal windowed sinusoidal template is given by [14]

(4) $$\begin{array}{c} v\left(t\right)=\cos\left(\omega_{c}\left(t\right)\right), \end{array}$$

where 0 ≤ *t* ≤ *T*, *T* is the window length, and *ω*_*c*_ is the carrier frequency. The normalized cross correlation function of the received pulse and windowed sinusoidal template can be calculated as [14]

(5) $$\begin{array}{c} R_{pv}\left(\tau \right)=\frac{1}{\sqrt{E_{p}}\sqrt{E_{v}}}\int_{-T/2}^{T/2}p\left(t\right)\cos\left(\omega_{c}\left(t-\tau \right)\right)dt, \end{array}$$

where *E*_*p*_ and *E*_*v*_ are the pulse and template energies, respectively. Assuming that the received pulse is the Gaussian pulse *p*(*t*) = *ω*_0_(*t*), this gives [10, 15, 16]

(6) Rpv(τ)=14EpEv[erf⁡(122σΦ)+erf⁡(122σΦ∗)] ×[exp⁡(−ωc2Λ)+exp⁡(−ωc2Λ∗)],

where Φ = *T* + 2*iω*_*c*_ *σ* ^2^, Λ = *σ* ^2^ *ω*_*c*_ + 2*iτ*, $i=\sqrt{-1}$, *ω*_*c*_ is the oscillator angular frequency in rad/sec, *T* is the window duration, *erf*⁡(·) is the error function defined as $\mathrm{erf}(x)=\left(2/\sqrt{\pi }\right)\int_{0}^{x}e^{-t^{2}}dt$, and *τ* is the time shift.

### 2.2. Suboptimal Square Templates

For a square template pulse *v*(*t*) = *A* rect  (*t*/*T*_*r*_) with amplitude *A* volts and duration *T*_*r*_, the normalized cross correlation function of the received and square template pulses can be calculated as

(7) $$\begin{array}{c} R_{pv}\left(\tau \right)=\frac{A}{\sqrt{E_{p}}\sqrt{E_{v}}}\int_{-T/2}^{T/2}p\left(t\right)\text{rect}\left(\frac{t-\tau }{T_{r}}\right)dt. \end{array}$$

Without loss of generality, we assume that the received pulse is the Gaussian pulse *p*(*t*) = *ω*_0_(*t*) and that the square pulse width is equal to *T*_*r*_ = *T*_*p*_ = 2*τ*_*p*_; this gives

(8) Rpv(τ)=Aτp22EpEv[erf⁡(2πτp(τ+2τp))] −Aτp22EpEv[erf⁡(2πτp(τ−2τp))].

**Figure 2:**
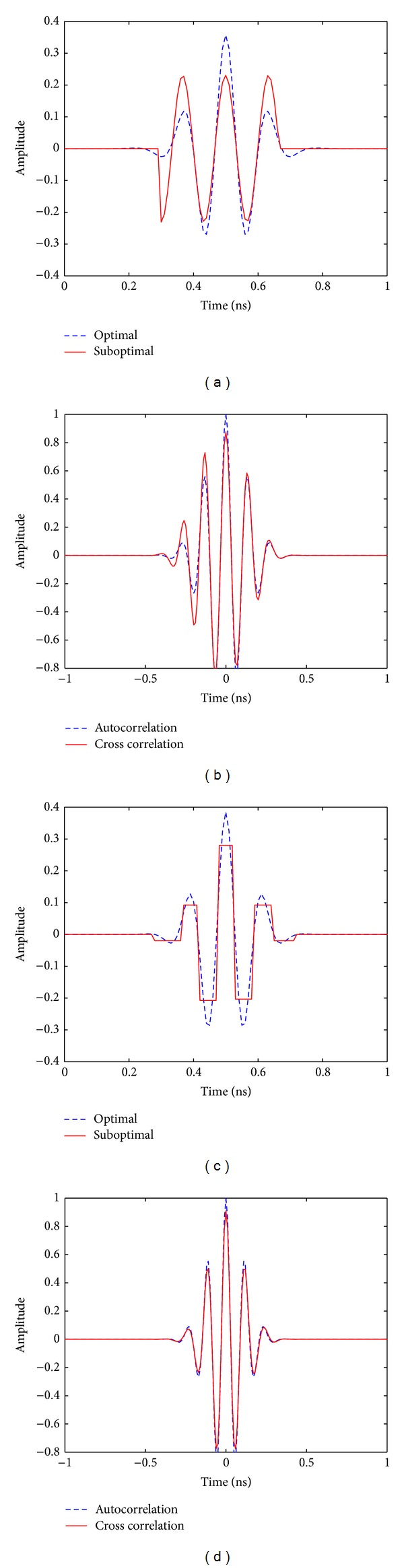
(a) Eighth-order Gaussian pulse and the corresponding suboptimal sinusoidal template. (b) Eighth-order Gaussian pulse autocorrelation and cross correlation with the corresponding suboptimal sinusoidal template. (c) Eighth-order Gaussian pulse and the corresponding suboptimal square pulse template. (d) Eighth-order Gaussian pulse autocorrelation and cross correlation with the corresponding suboptimal square pulse template.

Figure 2(a) shows the eighth-order Gaussian pulse and suboptimal sinusoidal template. The received pulse autocorrelation and cross correlation with sinusoidal template are shown in Figure 2(b). As can be seen, the pulse and autocorrelation function are well approximated by the suboptimal template and cross correlation function, respectively. Similarly, Figure 2(c) shows the optimal and suboptimal square pulse templates assuming the eighth-order Gaussian pulse and Figure 2(d) shows the corresponding autocorrelation and cross correlation functions.

## 3. BER Performance Analysis

For MRC detection at the destination node, the moment-generating functions (MGF) in terms of the approximate SNR is given by [1, 19]

(9) $$\begin{array}{c} M_{\gamma_{b}}\left(s\right)=M_{\gamma_{f}}\left(s\right)\prod_{i=1}^{L}M_{\gamma_{i}}\left(s\right), \end{array}$$

where *γ*_*f*_ is the instantaneous SNR between the source and destination, *L* is the number of combined paths, *γ*_*b*_ is the upper bound on the total SNR, and *M*_*γ*_*b*__ and *M*_*γ*_*f*__ are the MGF of *γ*_*b*_ and *γ*_*f*_, respectively. *M*_*γ*_*i*__ is calculated as on (11) [1, 19], and *M*_*γ*_*f*__ is equal to [1, 20]

(10) $$\begin{array}{c} M_{\gamma_{f}}\left(s\right)={\left(1+\frac{{\overset{-}{\gamma }}_{f}}{m_{f}}s\right)}^{-m_{f}}, \end{array}$$

where *m*_*f*_ and *m*_*g*_ are the Nakagami-*m* fading parameters of the links between the source to relay and relay to destination, respectively. Γ(·, ·) is the incomplete gamma function, Γ(·) is the gamma function, and _2_ *F*_1_(·, ·, ·) is the Gauss' hypergeometric function [1, 20]. Consider

(11) Mγi(s)=(mhiγ−hi)mhi(mgiγ−gi)mgiΓ(mhi+mgi)Γ(mhi)Γ(mgi) ×MM×1(mgi/γ−gi+mhi/γ−hi+s)mgi+mhi,

where

(12) MM=[1mhiF21(1,mhi+mgi;mhi+1;mhi/γ−hi+smhi/γ−hi+mgi/γ−gi+s)+1mgiF21(1,mhi+mgi;mgi+1;mgi/γ−gi+smhi/γ−hi+mgi/γ−gi+s)].

If *m*_*h*_*i*__ = *m*_*g*_*i*__ = *m*_*i*_ and ${\overset{-}{\gamma }}_{h_{i}}$= ${\overset{-}{\gamma }}_{m_{i}}$=*ρ*_*i*_, then (11) can be simplified to [1]

(13) Mγi(s)=(miρi)2miΓ(2mi)miΓ2(mi)2((2mi/ρi)+s)2mi ×F12(1,2mi;mi+1;(mi/ρi)+s(2mi/ρi)+s).

Substituting (10) and (11) in (9), this gives a closed form of *M*_*γ*_*b*__. In the following analysis, we will obtain the BER of EC-PPM scheme based on the calculated MGF. In BPPM, with a transmitted pulse *p*(*t*), the optimal template is calculated as in [21]

(14) $$\begin{array}{c} v\left(t\right)=p\left(t\right)-p\left(t-\delta \right), \end{array}$$

where *δ* is the PPM modulation parameter. In the case of the optimum receiver, the BER can be minimized by choosing *δ* to minimize the autocorrelation [22] as follows:

(15) $$\begin{array}{c} \delta_{\text{opt}}=\arg\left\{\underset{\delta }{\min}\;R_{pp}\left(\delta \right)\right\}. \end{array}$$

### 3.1. Performance in Additive White Gaussian Noise (AWGN) Channel

For *M*-ary EC-PPM, the transmitted signal is composed of *N*_*s*_ time-shifted pulses with 2 ≤ *M* < *N*_*s*_, where each signal is identified by a sequence of cyclic shifts of an *m*-sequence of length *N*_*s*_ [22]. The union bound on the bit error probability of *M*-ary EC-PPM assuming an optimum receiver is [22]

(16) $$\begin{array}{c} U_{BPb}=\frac{M}{2}Q\left(\sqrt{\frac{E_{s}}{2N_{0}}\left(1-R_{pp\min}\right)}\right), \end{array}$$

where Q(·) is the Gaussian tail function [20, 22]. The alternate representation for the tail function is expressed as *Q*(*x*) = (1/*π*)∫_0_^*π*^exp⁡(−(*x* ^2^/2sin^2^(*θ*)))*dθ* [20], *R*_*pp*min⁡_≜*R*_*pp*_(*δ*_opt_), and *E*_*p*_ is the pulse energy. To minimize BER, we wish to choose the value of *δ* that minimizes the correlation *R*_*pv*min⁡_(*δ*_opt_). Further, at the receiver, we choose a sample time *μ* to maximize the correlation between the suboptimal template and the generated pulse [12, 17] as follows:

(17) $$\begin{array}{c} \mu_{\text{opt}}=\arg\left\{\underset{\mu }{\max}R_{pv}\left(\mu \right)\right\}, \end{array}$$

with *R*_*pv*max⁡_ = *R*_*pv*_(*μ*_opt_); the union bound on the bit error probability for equally correlated signals is defined as [10, 15]

(18) $$\begin{array}{c} U_{BPb}=\frac{M}{2}Q\left(\sqrt{\frac{E_{s}}{2N_{0}}\left(R_{pv\max}-R_{pv\min}\right)}\right). \end{array}$$

### 3.2. Performance in Dense Multipath Channels

The BER of low complexity Partial Rake (PRake) receivers [23, 24], assuming PPM modulation and optimal templates in terms of MGF, *M*_*η*_*l*__, over a Nakagami-*m* channel with uniform power delay profile (PDP), and *L*_*p*_ independent identically distributed (i.i.d.) paths, is [23, 25]

(19) $$\begin{array}{c} P_{b,\text{PRake}}=\frac{1}{\pi }\int_{0}^{\pi /2}{\left(M_{\eta_{l}}\left(-\frac{\left(1-R_{pp\min}\right)}{4m\;\text{si}{\text{n}}^{2}\theta }\right)\right)}^{L_{\text{p}}}d\theta , \end{array}$$

where $\overset{-}{\eta }=E_{s}/LN_{0}$. Ideal Rake (ARake) receivers capture all the energy in all *L* paths; that is, *L*_*p*_ = *L* [23]. Substituting with the MGF $M_{\overset{-}{\eta }}(s)={\left(1-s\overset{-}{\eta }/m\right)}^{-m}$ gives [10, 15]

(20) $$\begin{array}{c} P_{b,\text{PRake}}=\frac{1}{\pi }\int_{0}^{\pi /2}{\left(\frac{4m\;\text{si}{\text{n}}^{2}\theta }{4m\;\text{si}{\text{n}}^{2}\theta +\overset{-}{\eta }\left(1-R_{pp\min}\right)}\right)}^{mL_{\text{p}}}d\theta . \end{array}$$

The probability of bit error of PRake receivers for PPM modulation with a suboptimal template is [10, 15]

(21) $$\begin{array}{c} P_{b,\text{PRake}}=\frac{1}{\pi }\int_{0}^{\pi /2}{\left(\frac{4m\text{si}{\text{n}}^{2}\theta }{4m\text{si}{\text{n}}^{2}\theta +\overset{-}{\eta }\left(R_{pv\max}-R_{pv\min}\right)}\right)}^{mL_{\text{p}}}d\theta . \end{array}$$

## 4. Numerical Results

**Figure 3:**
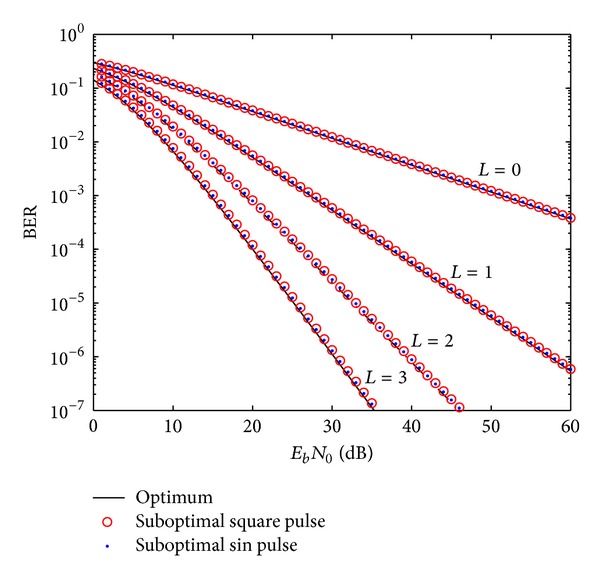
BER performance comparison of cooperative EC-BPPM modulation in Nakagami-*m* channel for the eighth order of the Gaussian pulse with optimal and suboptimal square templates.

In this section, we use the analysis provided in Section 3 to compare the performance of cooperative IR-UWB correlation receivers with the optimal and suboptimal templates.  Figure 3 shows a performance comparison of cooperative IR-UWB coherent receivers with optimal and suboptimal sinusoidal and square pulses. As can be seen, suboptimal pulses are traded for a minimal BER performance. Furthermore, one relay node provides ≈23 dB BER performance enhancement as compared to noncooperative communications at 1*e* − 3 BER.

This value is sufficient for mitigating the effect of attenuation caused by body-limb obstruction in NLOS links in WBSNs. Using two and three relay nodes further provides 9 and 4 dB performance enhancement for the same BER, respectively.

## 5. Conclusions

This paper studied the performance of cooperative IR-UWB suboptimal template-based detectors for green WBSNs. Results showed that suboptimal templates are traded for minimal BER performance degradation and that one relay node is sufficient for mitigating the effect of attenuation caused by body-limb obstruction in NLOS links.
